# Ectopic Adrenocorticotropic Hormone (ACTH)-Dependent Cushing Syndrome Secondary to Olfactory Neuroblastoma: A Rare Entity

**DOI:** 10.1016/j.aed.2025.08.004

**Published:** 2025-08-21

**Authors:** Naseem Eisa

**Affiliations:** Division of Endocrinology, Diabetes, and Metabolism, Department of Internal Medicine, Community Health Partners, Fresno, CA

**Keywords:** cushing syndrome, ectopic ACTH syndrome, neuroendocrine tumor, olfactory neuroblastoma, paraneoplastic syndrome

## Abstract

**Background/Objective:**

Ectopic adrenocorticotropic hormone (ACTH)-dependent Cushing syndrome is a rare paraneoplastic disorder caused by excessive cortisol production from nonpituitary tumors. Olfactory neuroblastoma (ONB), a rare neuroendocrine malignancy of the sinonasal cavity, is an exceedingly uncommon source of ectopic ACTH production, with fewer than 25 cases reported worldwide. This report presents a case of ACTH-dependent Cushing syndrome due to ONB, emphasizing the diagnostic complexity, multidisciplinary management, and favorable clinical outcomes.

**Case Presentation:**

A 70-year-old male presented with progressive muscle weakness, facial rounding, weight gain, hypertension, hypokalemia, and recurrent epistaxis. Laboratory evaluation revealed marked hypercortisolism and elevated plasma ACTH. Imaging demonstrated an expansile ethmoid sinus mass. Inferior petrosal sinus sampling excluded a pituitary source of ACTH. Endoscopic biopsy confirmed Hyams grade 2 ONB with positive immunohistochemical staining for neuroendocrine markers and ACTH. The patient received preoperative cortisol-lowering therapy and underwent complete endoscopic tumor resection followed by adjuvant radiotherapy. Postoperative assessment showed biochemical remission, resolution of Cushingoid features, and eventual recovery of the hypothalamic–pituitary–adrenal axis.

**Discussion:**

This case highlights the importance of a systematic diagnostic approach that includes biochemical testing, imaging, inferior petrosal sinus sampling, and histopathology to identify ectopic ACTH sources. It demonstrates the necessity of collaboration among endocrinology, otolaryngology, neurosurgery, radiology, and oncology teams in managing rare ACTH-secreting tumors.

**Conclusion:**

Timely diagnosis and definitive surgical resection of ACTH-producing ONB, along with endocrine stabilization and adjuvant radiotherapy, can lead to endocrine remission and improved long-term outcomes.

**Highlights**

• Rare case of ectopic adrenocorticotropic hormone syndrome secondary to olfactory neuroblastoma
• Diagnostic challenges highlighted, including nondiagnostic inferior petrosal sinus sampling results
• Multidisciplinary approach enabled complete tumor resection and hormonal remission
• Preoperative ketoconazole minimized perioperative cortisol-related morbidity
• Adjuvant radiotherapy optimized local control in intermediate-risk olfactory neuroblastoma

**Clinical Relevance**

This case emphasizes the importance of recognizing olfactory neuroblastoma as a rare source of ectopic adrenocorticotropic hormone production. It demonstrates the value of integrated biochemical, radiologic, surgical, and histopathologic strategies to achieve endocrine remission and prevent recurrence.

## Introduction

Ectopic ACTH syndrome (EAS) is a rare paraneoplastic disorder resulting in ACTH-dependent hypercortisolism, which manifests clinically as Cushing syndrome. Although it accounts for approximately 10% to 15% of ACTH-dependent cases, EAS is most frequently caused by bronchial carcinoids, small cell lung carcinoma, and pancreatic neuroendocrine tumors.^1^^,^^2^ In contrast, olfactory neuroblastoma (ONB), also known as esthesioneuroblastoma—a neuroendocrine malignancy of the upper nasal cavity—is a highly uncommon cause, with fewer than 1% of ONB cases associated with EAS.^2^^,^^3^

ONB arises from the olfactory epithelium and represents 2% to 3% of all sinonasal cancers.^4^^,^^5^ Its nonspecific presentation—ranging from nasal obstruction to epistaxis or anosmia—can delay diagnosis, and advanced tumors may invade adjacent structures such as the orbit or anterior cranial fossa.^4^^,^^5^ Histological overlap with other small round blue cell tumors necessitates immunohistochemical markers such as synaptophysin, chromogranin A, and S-100 for accurate identification.^4^^,^^6^ Factors such as age may influence tumor behavior, treatment selection, and prognosis.^7^

When ONB presents with ectopic ACTH secretion, the resulting hypercortisolism can lead to profound metabolic and cardiovascular complications.^8^^,^^9^ Due to its extreme rarity, this combination may not be initially suspected, delaying targeted therapy. This report presents a rare case of ACTH-dependent Cushing syndrome caused by ONB, highlighting the diagnostic complexity and need for multidisciplinary management.^3^^,^^10^

## Case Presentation

A 70-year-old male presented with 6 weeks of progressively worsening generalized, proximal muscle weakness, intermittent headaches, recurrent nosebleeds, abdominal fullness, leg swelling, and an unexplained 20-pound (9.1 kg) weight gain.

His medical history includes asthma, benign prostatic hyperplasia, hyperlipidemia, and retained shrapnel in the neck from military service in Vietnam. He has no history of hypertension, diabetes, or smoking. His family history includes a father who suffered a myocardial infarction at 51 years old, a mother with rheumatoid arthritis and osteoporosis, and a maternal uncle with lupus. His current medications include rosuvastatin 5 mg daily, tamsulosin 0.4 mg daily, and an albuterol inhaler as needed.

On examination, his vital signs were notable for an elevated blood pressure of 171/84 mmHg (normal: <120/<80 mmHg), a temperature of 37.2 C (99 F) (normal: 36.1–37.2°C [97–99 F]), a heart rate of 91 bpm (normal: 60–100 bpm), a respiratory rate of 16 breaths per minute (normal: 12–20 breaths per minute), an oxygen saturation of 92% on room air (normal: ≥95%), and a weight of 78.9 kg (174 lb). Physical examination revealed a round plethoric face ("moon facies,") a prominent dorsocervical fat pad ("buffalo hump,") supraclavicular fullness, mild abdominal tenderness, violaceous striae across the abdomen, diffuse soft tissue swelling, and bilateral 2+ pitting edema in the lower extremities.

### Diagnostic Assessment

Laboratory evaluation demonstrated severe hypokalemia (1.6 mEq/L [1.6 mmol/L]; normal: 3.5–5.0 mEq/L [3.5–5.0 mmol/L]) and marked fasting hyperglycemia (244.0 mg/dL [13.5 mmol/L]; normal: 70–99 mg/dL [3.9–5.5 mmol/L]), in addition to leukocytosis, hypochloremia, acute kidney injury, hypoproteinemia, and hypoalbuminemia.

Hormonal evaluation (Table 1) was consistent with ACTH-dependent hypercortisolism, characterized by elevated serum cortisol and ACTH concentrations, lack of suppression with dexamethasone, and suppressed dehydroepiandrosterone sulfate (DHEA-S). Aldosterone and plasma renin activity were within normal limits, effectively excluding primary hyperaldosteronism. Plasma free metanephrines and normetanephrines were also within reference ranges, ruling out pheochromocytoma. Repeat morning cortisol remained markedly elevated, and late-night salivary cortisol levels on 2 occasions were significantly above the reference range. Twenty-four-hour urinary free cortisol (UFC) was profoundly elevated on both collections. Following a 1 mg overnight dexamethasone suppression test, serum cortisol, ACTH, and dexamethasone levels confirmed a lack of cortisol suppression despite adequate dexamethasone absorption (Table 1). These results were consistent with ACTH-dependent Cushing syndrome.

**Table 1:** Hormone Panel Results

Test	Value	Normal Range
AM cortisol	29 μg/dL (800.11 nmol/L) (high)	3.7–19.4 μg/dL (102–535 nmol/L)
Repeated AM cortisol	26 μg/dL (717.34 nmol/L) (high)	3.7–19.4 μg/dL (102–535 nmol/L)
ACTH	250 pg/mL (30.03 pmol/L) (high)	10–60 pg/mL (2.2–13.2 pmol/L)
Plasma renin activity	1.2 ng/mL/h (1.2 μg/L/h) (normal)	0.2–4.0 ng/mL/h (0.2–4.0 μg/L/h)
DHEA-S	50 μg/dL (1.25 μmol/L) (low)	65–380 μg/dL (1.75–10.26 μmol/L)
Aldosterone, blood	4. 9 ng/dL (0.14 nmol/L) (normal)	4.0–31.0 ng/dL (110–860 pmol/L)
Plasma free metanephrines	0.34 nmol/L (0.034 μg/L) (normal)	<0.50 nmol/L (<0.09 μg/L)
Plasma free normetanephrines	0.75 nmol/L (0.075 μg/L) (normal)	<0.90 nmol/L (<0.16 μg/L)
Late-night salivary cortisol (1st)	0.27 μg/dL (7.45 nmol/L) (high)	≤0.09 μg/dL (≤2.5 nmol/L) (10 PM–1 AM)
Late-night salivary cortisol (2nd)	0.36 μg/dL (9.93 nmol/L) (high)	≤0.09 μg/dL (≤2.5 nmol/L) (10 PM–1 AM)
24-h urinary free cortisol (1st)	5880.0 μg/d (16 223 nmol/d) (high)	≤60.0 μg/d (≤165 nmol/d)
24-h urinary free cortisol (2nd)	4920.0 μg/d (13 576 nmol/d) (high)	≤60.0 μg/d (≤165 nmol/d)
AM cortisol level (after 1 mg dexamethasone)	12.3 μg/dL (339 nmol/L) (high)	<1.8 μg/dL (<50 nmol/L) adequate suppression
Dexamethasone level(after 1 mg dexamethasone)	336 ng/dL (8.64 nmol/L) (normal)	>200 ng/dL (>5.2 nmol/L) adequate absorption
ACTH level (after 1 mg dexamethasone)	242 pg/mL (53.27 pmol/L) (not suppressed)	10–60 pg/mL (2.2–13.2 pmol/L)

Abbreviations: μg/d = micrograms per day; μg/dL = Micrograms per deciliter; μg/L = micrograms per liter; μmol/L = micromoles per liter; AM = morning (Ante Meridiem); nmol/L = nanomoles per Liter; ng/mL/h = nanograms per milliliter per hour; pmol/L = picomoles per liter; pg/mL = picograms per milliliter; μg/L/h = micrograms per liter per hour; ng/dL = nanograms per deciliter; nmol/d = nanomoles per day.

Inferior petrosal sinus sampling (IPSS) was performed using contrast-enhanced fluoroscopy to confirm accurate catheter placement in both inferior petrosal sinuses. Absolute ACTH values obtained during IPSS are shown in (Table 2). The central-to-peripheral ACTH gradient at baseline was 1.1, which is below the diagnostic threshold of 2.0 typically required to support a pituitary source of ACTH. Following desmopressin acetate (DDAVP) stimulation, peak left: peripheral and right: peripheral ACTH ratios reached 1.7 and 1.5, respectively—well below the accepted post-stimulation cut-off of 3.0. In addition, the left: right petrosal ACTH ratios remained between 1.03 and 1.15 throughout the sampling period, indicating no significant lateralization of ACTH secretion. These findings are not consistent with Cushing’s disease and instead support a diagnosis of ectopic ACTH syndrome.

**Table 2:** Bilateral Petrosal Sinus and Peripheral Adrenocorticotropin Levels Before and After Intravenous Injection of Desmopressin Acetate (DDAVP) 10 mcg

Time post DDAVP, min	Left petrosal ACTH	Left: peripheral ACTH	Right petrosal ACTH	Right: peripheral ACTH	Peripheral ACTH	Left: right petrosal ACTH
0	165 pg/mL (36.3 pmol/L)	1.1	160 pg/mL (35.2 pmol/L)	1.1	150 pg/mL (33.0 pmol/L)	1.03
3	270 pg/mL (59.4 pmol/L)	1.6	245 pg/mL (53.9 pmol/L)	1.4	170 pg/mL (37.4 pmol/L)	1.10
5	320 pg/mL (70.4 pmol/L)	1.7	285 pg/mL (62.7 pmol/L)	1.5	185 pg/mL (40.7 pmol/L)	1.12
10	350 pg/mL (77.0 pmol/L)	1.4	305 pg/mL (67.2 pmol/L)	1.2	250 pg/mL (55.0 pmol/L)	1.15

Abbreviations: ACTH = adrenocorticotropin; DDAVP = desmopressin acetate; pg/mL = picograms per milliliter; pmol/L = picomoles per liter.

**Fig. 1:**
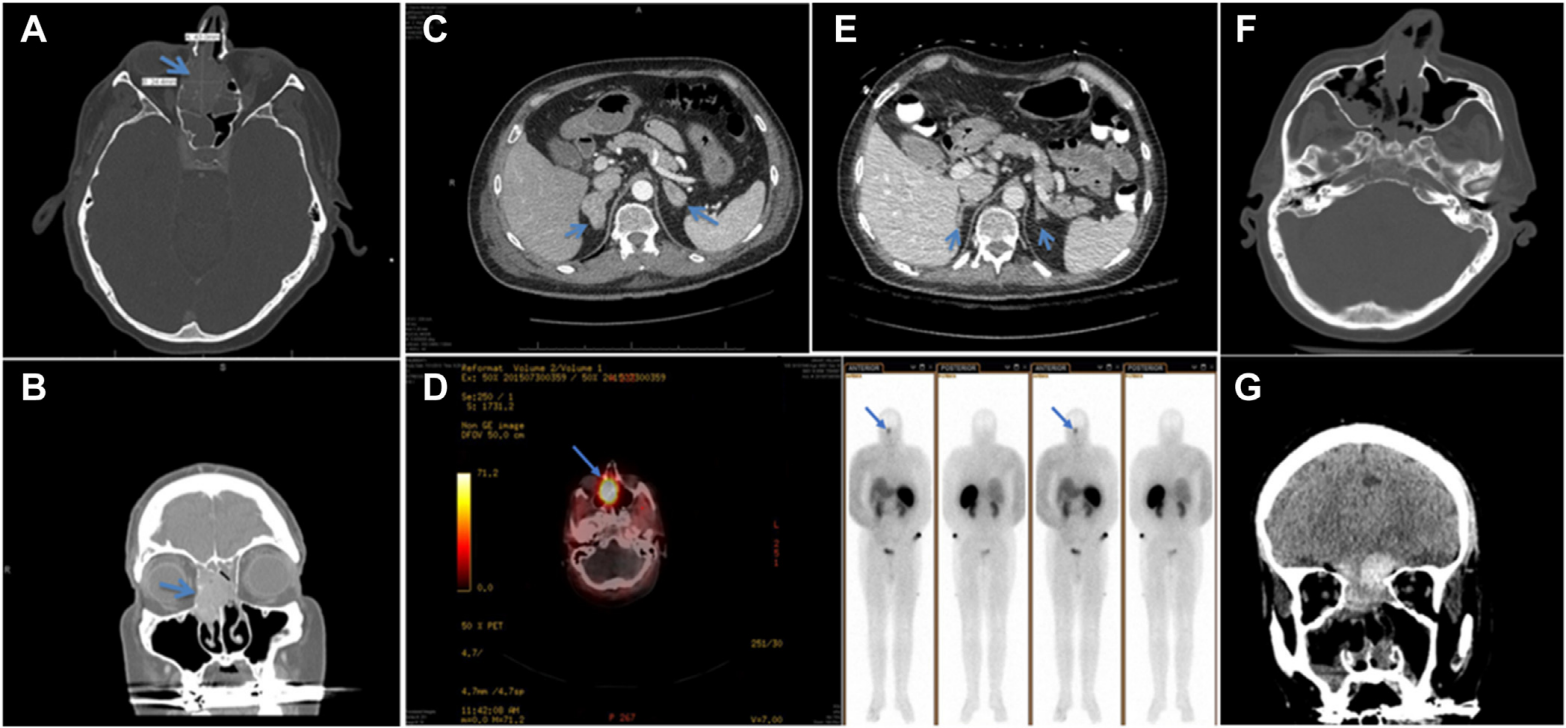
(*A*) Axial and (*B*) coronal noncontrast computed tomography (CT) images of the head demonstrate a heterogeneous soft tissue mass at the anterior skull base extending toward the cribriform plate and into the *right* nasal cavity, involving the ethmoid sinus and eroding the lamina papyracea, resulting in medial displacement of the *right* orbital contents (blue arrows). (*C*) Axial contrast-enhanced CT of the abdomen reveals bilateral adrenal gland enlargement. (*D*) Whole-body single-photon emission computed tomography/computed tomography (SPECT/CT) using indium-111 pentetreotide demonstrates intense radiotracer uptake localized to the biopsy-confirmed esthesioneuroblastoma in the ethmoid sinuses, with no evidence of metastatic octreotide-avid lesions. (*G*) Coronal contrast-enhanced CT scan of the abdomen, performed after surgery, shows normalization in the size of both adrenal glands. (*E*) Coronal and (*F*) axial noncontrast CT images of the paranasal sinuses obtained postoperatively demonstrate complete surgical resection of the tumor.

Magnetic resonance imaging of the head could not be performed due to a history of retained shrapnel in the neck from combat in Vietnam. Noncontrast computed tomography (CT) images of the head and paranasal sinuses revealed no evidence of a pituitary tumor but demonstrated an expansile mass measuring approximately 2.4 × 4.3 × 3.3 cm, centered within the bilateral ethmoid sinuses with extension into both the anterior and posterior ethmoidal air cells (Fig. 1*A, B*). A contrast-enhanced CT scan of the abdomen, performed following improvement in renal function, demonstrated marked bilateral adrenal gland enlargement (Fig. 1*C*).

**Fig. 2:**
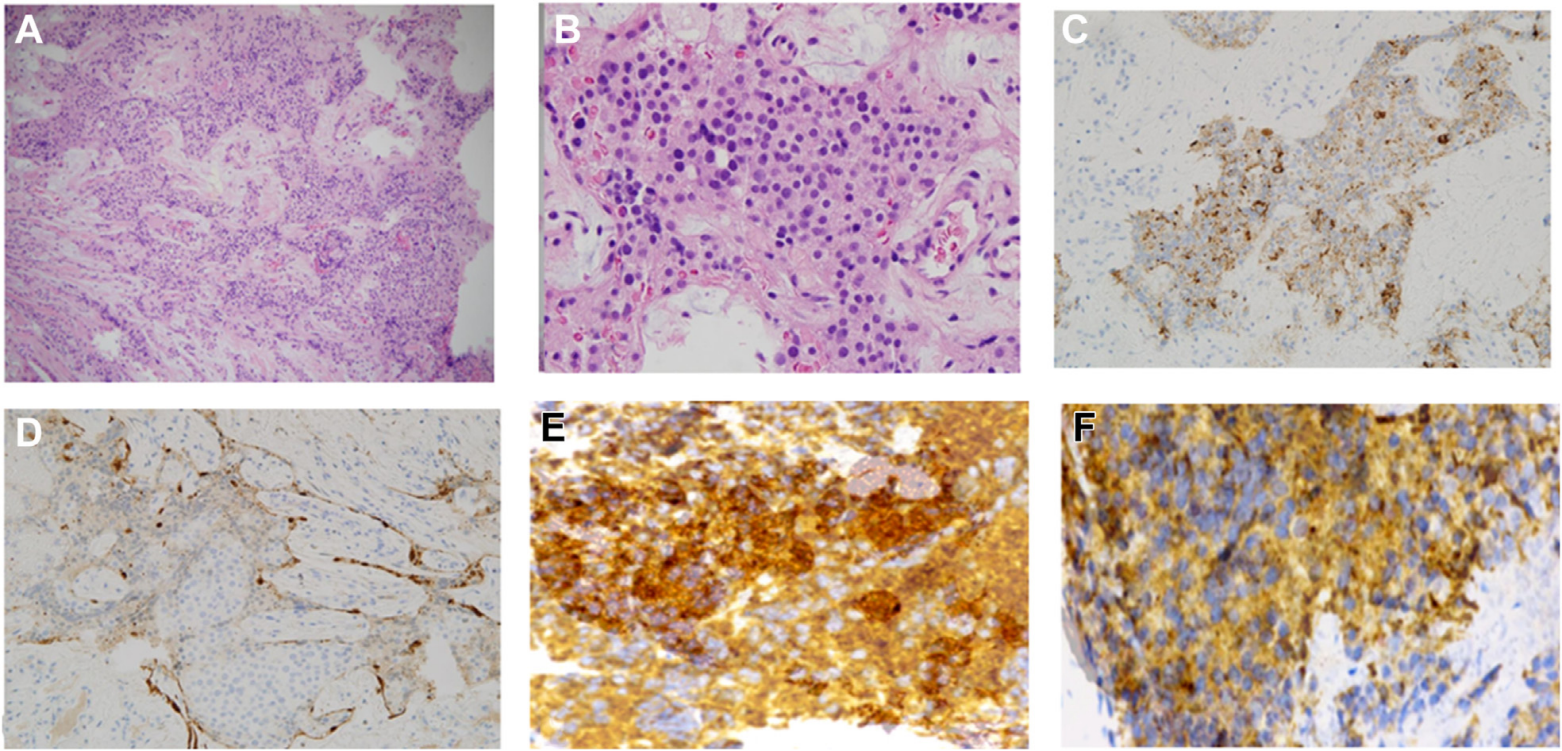
(*A*) Low-power H&E (4×) shows well-circumscribed lobules of small round blue cells with fibrovascular stroma and a neurofibrillary matrix; no necrosis or rosettes are seen. (*B*) High-power H&E (40×) reveals neoplastic cells with high nuclear-to-cytoplasmic ratio, hyperchromatic nuclei, and granular chromatin, consistent with Hyams Grade 2 ONB. (*C*) Chromogranin A shows granular cytoplasmic positivity in tumor nests, confirming neuroendocrine differentiation. (*D*) Synaptophysin shows diffuse granular cytoplasmic staining in tumor clusters, with negative stromal background. (*E*) S-100 highlights sustentacular cells in a peripheral pattern around tumor nests. (*F*) ACTH staining shows patchy to diffuse cytoplasmic positivity in tumor cells, confirming ectopic ACTH production in ONB. A nuclear medicine octreotide scan (111 Indium-pentetreotide scintigraphy) with single-photon emission computed tomography/computed tomography (SPECT/CT) demonstrated intense radiotracer uptake in the biopsy-proven esthesioneuroblastoma centered within the ethmoid sinuses, confirming the tumor’s expression of somatostatin receptors. There was no evidence of locoregional or distant metastatic disease demonstrating octreotide avidity (Fig. 1*D*).

The otolaryngology (ENT) team was consulted and recommended an endoscopic biopsy of the nasal mass. Histopathologic examination revealed a Hyams Grade 2 olfactory neuroblastoma (Fig. 2*A, B*), characterized by well-circumscribed lobules of small round blue cells with scant cytoplasm, a neurofibrillary background matrix, and low mitotic activity, without necrosis or rosette formation—findings typical of a moderately differentiated tumor in the Hyams grading system.

Immunohistochemical staining supported the diagnosis: tumor cells were positive for chromogranin A (Fig. 2*C*), synaptophysin (Fig. 2*D*), and S-100 (Fig. 2*E*). Chromogranin A and synaptophysin are markers of neuroendocrine differentiation, confirming the tumor’s neuroendocrine origin. S-100 positivity in the sustentacular cells surrounding tumor nests is a classic feature of olfactory neuroblastoma. Staining was negative for neurofilament protein, AE1/AE3, and epithelial membrane antigen, helping exclude other small round blue cell tumors, such as neuroendocrine carcinoma or sinonasal undifferentiated carcinoma. Importantly, the tumor cells showed positive cytoplasmic staining for ACTH (Fig. 2*F*), confirming ectopic ACTH production by the tumor itself. This finding definitively links the olfactory neuroblastoma as the source of paraneoplastic ACTH secretion, consistent with the patient’s clinical picture of ectopic Cushing's syndrome.

### Treatment

Hypokalemia was corrected, and oral ketoconazole 200 mg twice daily was initiated preoperatively to mitigate the metabolic complications of hypercortisolism. Ketoconazole was discontinued on the day of surgery. The tumor was resected via an endoscopic endonasal approach. A blood sample was obtained immediately following tumor removal for measurement of ACTH and cortisol levels. Intravenous hydrocortisone (100 mg every 6 h) was initiated promptly thereafter. Postoperative cortisol and ACTH levels were undetectable: cortisol <5 μg/dL [<138 nmol/L] (normal: 5–25 μg/dL [138–690 nmol/L]); ACTH <5 pg/mL [<1.1 pmol/L] (normal: 10–60 pg/mL [2.2–13.3 pmol/L]). These findings confirmed successful surgical resection of the ACTH-secreting tumor. These issues extended the hospital stay and required treatment with antiseizure medications, antibiotics, and additional surgeries by ENT and Neurosurgery teams.

### Outcome and Follow-Up

The patient demonstrated significant normalization of blood pressure (124/78 mmHg), fasting blood glucose (95 mg/dL [5.3 mmol/L]), and potassium (4.3 mEq/L [4.3 mmol/L]) within 2 weeks postoperatively. ACTH levels decreased from preoperative values of 220–250 pg/mL (48.4–55.2 pmol/L) to 29 pg/mL (5.5 pmol/L), and morning (AM) cortisol levels decreased from preoperative values of 29 μg/dL (800 nmol/L) to 12 μg/dL (331 nmol/L). These values were obtained at 2 weeks postoperatively. While early normalization of ACTH and cortisol levels could raise concern for residual disease, the patient’s subsequent sustained biochemical remission, clinical recovery, and a robust response to cosyntropin stimulation at 3 months post-op were reassuring. Adjuvant radiotherapy was also administered to mitigate any potential risk of recurrence.

He was subsequently transferred to an inpatient rehabilitation facility while receiving oral hydrocortisone replacement therapy, during which his functional status progressively improved. The patient was later discharged home on oral hydrocortisone replacement therapy with plans for continued outpatient physical therapy. Hydrocortisone was gradually tapered and discontinued 3 months after surgery, at which point blood pressure (122/76 mmHg), fasting glucose (90 mg/dL [5.0 mmol/L]), potassium (4.2 mEq/L [4.2 mmol/L]), ACTH (25 pg/mL [4.9 pmol/L]), and AM cortisol (15 μg/dL [414 nmol/L]) demonstrated sustained normalization. Following administration of 250 mcg intramuscular cosyntropin, serum cortisol peaked at 21 μg/dL (580 nmol/L), confirming an adequate adrenal reserve and complete recovery of the hypothalamic–pituitary–adrenal axis. Additionally, late-night salivary cortisol was remeasured on 2 occasions after hydrocortisone discontinuation and found to be 0.04 μg/dL (1.10 nmol/L) and 0.03 μg/dL (0.83 nmol/L), both within normal reference limits (≤0.09 μg/dL [≤2.5 nmol/L]). A 24-hour UFC collected at the same time measured 38 μg/d (105 nmol/d), confirming biochemical resolution of hypercortisolism. Cushing’s stigmata, including muscle weakness and skin changes, showed marked improvement by 3 months postoperatively (Table 3).

**Table 3:** Timeline of Clinical and Biochemical Recovery Following Resection of Ectopic ACTH-Secreting Olfactory Neuroblastoma

Parameter	Preoperative value	24–48 h Postop	2 wks postop	3 mo postop	Normal range
Blood pressure	171/84 mmHg	140/80 mmHg	124/78 mmHg	122/76 mmHg	<130/80 mmHg
Fasting glucose	244 mg/dL (13.5 mmol/L)	160 mg/dL (8.9 mmol/L)	95 mg/dL (5.3 mmol/L)	90 mg/dL (5.0 mmol/L)	70–99 mg/dL (3.9–5.5 mmol/L)
Potassium	1.6 mEq/L (1.6 mmol/L)	3.8 mEq/L (3.8 mmol/L)	4.3 mEq/L (4.3 mmol/L)	4.2 mEq/L (4.2 mmol/L)	3.5–5.0 mEq/L (3.5–5.0 mmol/L)
ACTH	220–250 pg/mL (48.4–55.2 pmol/L)	<10 pg/mL (<2.2 pmol/L)	29 pg/mL (5.5 pmol/L)	25 pg/mL (4.9 pmol/L)	10–60 pg/mL (2.2–13.3 pmol/L)
AM cortisol	29 μg/dL (800 nmol/L)	<5 μg/dL (<138 nmol/L)	12 μg/dL (331 nmol/L)	15 μg/dL (414 nmol/L); Cosyntropin peak: 21 μg/dL (580 nmol/L)	5–25 μg/dL (138–690 nmol/L); adequate response >18 μg/dL (500–550 nmol/L)
LNSC	0.27/0.36 μg/dL (7.45/9.93 nmol/L)	—	—	0.04/0.03 μg/dL (1.10/0.83 nmol/L)	≤0.09 μg/dL (≤2.5 nmol/L) (10 PM–1 AM)
UFC (24-h)	5880/4920 μg/d (16 223/13 576 nmol/d)	—	—	38 μg/d (105 nmol/d)	≤60 μg/d (≤165 nmol/d)
Cushing’s Stigmata	Moon facies, dorsocervical fat pad, violaceous striae, severe muscle weakness	No change	Partial improvement: BP/glucose control; decreased edema	Marked improvement; muscle strength restored; striae fading	Not applicable

Abbreviations: ACTH = adrenocorticotropin; mmHg = illimeters of mercury; mEq/L = milliequivalents per liter; mg/dL = milligrams per deciliter; mmol/L = millimoles per liter; μg/dL = micrograms per deciliter; AM = morning (Ante Meridiem); pg/mL = picograms per milliliter; pmol/L = picomoles per liter; nmol/L = nanomoles per liter.

dfA follow-up CT scan of the adrenals with contrast, performed following improvement in renal function, confirmed normalization in the size of the previously enlarged adrenal glands (Fig. 1*E*). A follow-up CT of sinuses without contrast confirmed complete resection of the tumor (Fig. 1*F, G*).

Adjuvant radiotherapy was recommended in view of the patient’s Kadish stage B tumor, Hyams grade 2 histology, and the elevated risk of local recurrence inherent to olfactory neuroblastoma. Despite complete surgical excision, radiotherapy was pursued to mitigate recurrence risk, particularly considering the tumor’s ectopic ACTH secretion, which suggested biologically aggressive behavior, as well as the patient’s satisfactory functional status and anticipated favorable treatment tolerance. A total of 30 fractions of 2 Gy were administered using volumetric modulated arc therapy.

## Discussion

### Diagnostic Considerations

EAS poses a significant diagnostic challenge due to its variable presentation and the urgency of identifying the source of ACTH excess. ONB, although rare, should be considered in patients with ACTH-dependent Cushing syndrome who present with sinonasal masses. ONB accounts for only 2% to 3% of all malignant sinonasal tumors,^4^^,^^6^ with fewer than 25 cases documented as sources of ectopic ACTH production.^3^^,^^11^^,^^12^

While ectopic ACTH syndrome remains the most well-recognized endocrine manifestation of ONB, a broader spectrum of paraneoplastic syndromes has also been described. These include syndrome of inappropriate antidiuretic hormone secretion, paraneoplastic hypercalcemia—often mediated by parathyroid hormone–related protein—and catecholamine excess mimicking pheochromocytoma.^11^ These atypical presentations underscore the neuroendocrine complexity of ONB and the diagnostic challenges they pose.

Diagnosis involves biochemical confirmation of hypercortisolism using low-dose dexamethasone suppression, 24-hour UFC, late-night salivary cortisol, and plasma ACTH levels. Interestingly, despite markedly elevated ACTH levels, our patient exhibited a low DHEA-S concentration and a normal aldosterone level. This biochemical pattern supports previous observations that EAS may present with a dissociation in adrenal steroidogenesis. Chronic hypercortisolemia may suppress the zona reticularis,^13^ while ectopic ACTH-producing tumors may secrete aberrant precursors that preferentially stimulate glucocorticoid rather than androgen synthesis.^14^ Cortisol excess can also downregulate key enzymes such as 17,20-lyase and SULT2A1, thereby impairing DHEA-S production.^15^ Moreover, the rapid onset and severity of ectopic ACTH production may preclude the compensatory DHEA-S rise typically observed in pituitary-driven Cushing disease. Although cortisol excess is known to suppress the renin-angiotensin-aldosterone system, aldosterone levels may remain detectable in certain EAS cases, particularly in early-stage or physiologically variable presentations.^16^

Once ACTH-dependence is established, localization of the tumor becomes essential. IPSS, although considered the gold standard for distinguishing pituitary from ectopic ACTH sources, may yield inconclusive results in cases of ONB due to altered venous drainage pathways.^3^ Functional imaging with ^111^In-octreotide single-photon emission computed tomography/computed tomography or ^68^Ga-DOTATATE positron emission tomography/computed tomography facilitates localization of neuroendocrine tumors that express somatostatin receptors. Histopathologic confirmation using ACTH immunostaining and neuroendocrine markers such as chromogranin A, synaptophysin, and S-100 is essential to confirm diagnosis.

### Therapeutic Approach and Challenges

Surgical resection remains the cornerstone of management for ACTH-producing ONB.^9^ Endoscopic endonasal approaches are preferred when anatomically feasible due to their minimally invasive nature and favorable access to the anterior skull base. Preoperative pharmacologic inhibition of cortisol biosynthesis (utilizing ketoconazole, which was specifically selected for our patient, metyrapone, or etomidate) represents a critical intervention to attenuate hypercortisolism-related metabolic complications and minimize perioperative morbidity.^3^^,^^8^ Intraoperative glucocorticoid replacement should be administered following tumor resection to prevent adrenal insufficiency. Postoperative complications—such as cerebrospinal fluid leak or infection—require prompt multidisciplinary intervention.

Adjuvant radiotherapy is generally recommended for intermediate-to high-grade ONBs, even after gross total resection, given their aggressive behavior and high risk of recurrence. Volumetric modulated arc therapy delivers precise radiation doses while minimizing toxicity to adjacent structures.^5^^,^^9^ Platinum-based chemotherapy remains a therapeutic option in patients with unresectable or metastatic disease.^9^

Emerging therapeutic strategies include somatostatin receptor–directed theranostics. Zhi et al (2025) recently demonstrated the dual diagnostic and therapeutic potential of ^68^Ga-DOTATATE positron emission tomography/computed tomography imaging and ^177^Lu-DOTATATE peptide receptor radionuclide therapy in ONB, offering promising future directions for patients with advanced or somatostatin receptor–positive disease.^17^

### Prognosis and Future Directions

The prognosis of ONB is influenced by Kadish staging, Hyams histologic grading, and treatment strategy. Recurrence rates are reported to range from 30% to 60%,^9^^,^^18^ and 5-year survival rates vary from 45% to 80% depending on tumor grade, stage, and completeness of resection.^6^^,^^19^ Early detection, complete surgical resection, and multimodal therapy, including radiotherapy, are associated with improved outcomes. Lifelong follow-up with serial imaging and endocrine evaluation is essential to monitor for recurrence and late-onset adrenal insufficiency.^10^^,^^19^

Continued advancements in molecular imaging and targeted therapies, particularly those leveraging somatostatin receptor biology, may expand the therapeutic landscape for patients with recurrent or progressive ONB.

## Conclusion

This case highlights the importance of timely diagnosis, comprehensive biochemical and radiologic assessment, and coordinated multidisciplinary management in ACTH-producing ONB. In addition to surgery and preoperative endocrine stabilization, adjuvant radiotherapy and long-term surveillance are critical components of care. As somatostatin receptor–based imaging and theranostic therapies evolve, they offer exciting opportunities to individualize treatment in this rare but challenging neuroendocrine malignancy.

## Statement of Patient Consent

Written informed consent was obtained from the patient for publication of this case report and any accompanying images.

## Disclosure

The author has no conflict of interest to disclose.
